# Recognizing the biological barriers and pathophysiological characteristics of the gastrointestinal tract for the design and application of nanotherapeutics

**DOI:** 10.1080/10717544.2024.2415580

**Published:** 2024-10-15

**Authors:** Shan Li, Tianyu Wu, Jingfeng Wu, Wensheng Chen, Dinglin Zhang

**Affiliations:** a Department of Chemistry, College of Basic Medicine, Army Medical University (Third Military Medical University), Chongqing, China; b Department of Gastroenterology, Southwest Hospital, Army Medical University (Third Military Medical University), Chongqing, China; c Army 953 Hospital, Shigatse Branch of Xinqiao Hospital, Army Medical University (Third Military Medical University), Shigatse, Tibet Autonomous Region, China

**Keywords:** Nanotherapeutics, gastrointestinal tract, inflammatory bowel disease, *Helicobacter pylori* infection

## Abstract

The gastrointestinal tract (GIT) is an important and complex system by which humans to digest food and absorb nutrients. The GIT is vulnerable to diseases, which may led to discomfort or even death in humans. Therapeutics for GIT disease treatment face multiple biological barriers, which significantly decrease the efficacy of therapeutics. Recognizing the biological barriers and pathophysiological characteristics of GIT may be helpful to design innovative therapeutics. Nanotherapeutics, which have special targeting and controlled therapeutic release profiles, have been widely used for the treatment of GIT diseases. Herein, we provide a comprehensive review of the biological barrier and pathophysiological characteristics of GIT, which may aid in the design of promising nanotherapeutics for GIT disease treatment. Furthermore, several typical diseases of the upper and lower digestive tracts, such as *Helicobacter pylori* infection and inflammatory bowel disease, were selected to investigate the application of nanotherapeutics for GIT disease treatment.

## 1. Introduction

The gastrointestinal tract (GIT) is a complex system that is composed of different specialized organs, including the stomach, small intestine, and large intestine. The main functions of the GIT are the digestion of food and the absorption of water, electrolytes, and nutrients (Arévalo-Pérez et al., 2020). Each part of the GIT can be considered a unique physical environment with characteristic pH values, motility, digestive juices and microbiomes. For example, the stomach has a low pH, the small intestine has optimal drug absorption, and the colon is rich in microbiota. The function of the GIT is closely related to daily life. Lifestyle and diet affect the occurrence and development of gastrointestinal diseases (Ifrim Chen et al., 2019). Therefore, gastrointestinal diseases are common in the population. Gastrointestinal diseases have different symptoms depending on the cause of the disease. These include esophageal and swallowing disorders, gastric and peptic ulcer disease, inflammatory bowel disease (IBD), irritable bowel syndrome (IBS) and gastrointestinal tumors (Greenwood-Van Meerveld et al., 2017; Ranjbar et al., 2022). The GIT is affected by regional specific anatomic, histological and functional disorders. Moreover, pathological injury during different diseases increases the difficulty of treatment. *In vivo*, drugs are susceptible to structural changes, degradation or loss of activity due to adverse gastrointestinal conditions, including pH changes and proteolytic enzymes. Additionally, some drugs are excreted or absorbed throughout the body, resulting in systemic exposure and severe side effects (Volmer et al., 2018). Therefore, there is an urgent need for novel drug delivery systems that can effectively control drug release to target sites, enhance drug efficacy and reduce side effects.

Oral administration, intravenous injection, and rectal administration are usually employed as the methods of therapeutics administration for GIT disease treatment. Oral administration of therapeutics may be the best choice for treating these patients because it is painless and offers the possibility of self-administration; in turn, there is more flexibility in the dosage regimen, which improve patient compliance and the eliminate the need for sterile conditions for product manufacture, thus reducing production costs (Date et al., 2016). By this route, the environmental extremes in the GIT may affect the stability and delivery of therapeutics. In addition, intravenous injection of therapeutics improves the response time and bioavailability and reduces the initial metabolism of drugs in the liver. However, this method may be accompanied by greater systemic side effects and poor compliance due to pain and complex procedures (Yang & Merlin, 2019). Rectal administration of therapeutics is a good option for distal colon-and rectum-specific diseases and can reduce systemic exposure. However, poor drug retention and compliance remain problems, and leakage, retention, and bloating may occur (Frei et al., 2012). The GIT is both the site of absorption of oral therapeutics and the target of some gastrointestinal diseases, so interactions between therapeutics and the GIT are quite frequent. Consequently, a clear understanding of the viability of the GIT microenvironment will help identify strategies to overcome biological barriers in the GIT. Nanotherapeutics can overcome a variety of physical and biological barriers in the GIT via epithelium enhanced permeability and retention (epEPR) or ligand-mediated targeting (Zhang et al., 2017). With special targeting ability and controlled drug release profiles, nanotherapeutics are widely used for the treatment of GIT diseases, including *Helicobacter pylori* (*H. pylori*) infections, IBD, and gastrointestinal tumors (Frei et al., 2012).

Some reviews have focused on developing nanotherapeutics for the treatment of GIT diseases, such as IBD, gastric ulcers, or colorectal cancer (Lamprecht, 2010). Herein, we provide a comprehensive review of the pathophysiological characteristics and biological barriers of the GIT, which may help researchers design promising nanotherapeutics for the treatment of GIT diseases. In addition, the application of nanotherapeutics for the treatment of nonneoplastic GIT diseases (e.g. IBD and *H. pylori* infection) was thoroughly reviewed, because tumors have their own biological barriers and pathophysiological microenvironment.

## 2. Challenges of therapeutics and opportunities of nanotherapeutics in the GIT

**Figure 1.**
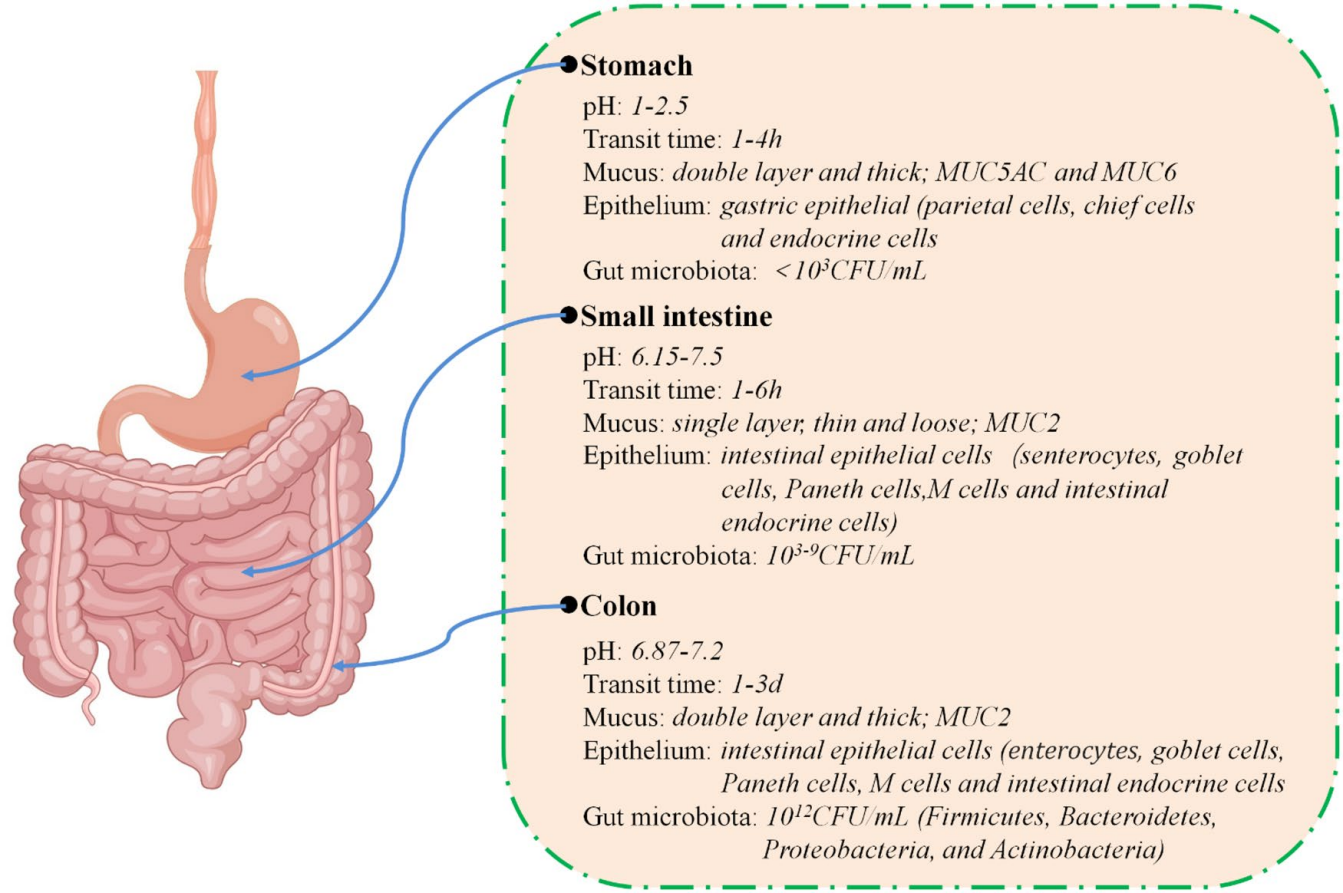
Physiological microenvironment of the gastrointestinal tract. Created by the author Shan Li.

The GIT has a complex physiological environment (Figure 1). For example, the pH of the stomach is 1.0-2.5, which increases to 6.15-7.50 in the small intestine, and remains at 6.87-7.20 in colon tissues. In addition, double layers and thick mucus are found in stomach and colon tissues. However, only single layers and thin and loose mucus are found in the small intestine (Figure 1). The category and number of gut microbiota in the stomach, small intestine, and colon were also different (Figure 1). The physiological environment changes during a morbid state. Potential nanotherapeutics should overcome the physiological barriers in the GIT or pass through blood circulation to reach the sites of disease to exert their activity; thus, there are great challenges in the treatment of GIT diseases.

Fortunately, a diseased GIT usually presents with an abnormal physiological microenvironment, which can be characterized by a low pH, a high concentration of reactive oxygen species (ROS), or overexpression of biomarkers; these characteristics provide an opportunity for the treatment of GIT diseases with nanotherapeutics. The opportunities and challenges associated with therapeutics for the treatment of GIT diseases are summarized in Table 1

**Table 1.** The opportunities and challenges of utilizing therapeutics for the treatment of GIT diseases.

Interfering factors	Challenges	Pathological changes	Potential nanotherapeutics for the treatment of GIT diseases
Biochemical-luminal barrier in GIT	Variable pH values	Reduced gastric pH values in hypergastrinemia Reduced colonic pH values in IBD	Preparation pH-responsive nanotherapeutics for controlled drug release
	Variable transit time	Reduced colonic transit time in irritable bowel syndrome, active ulcerative colitis	Fabrication time-responsive nanotherapeutics or pressure-responsive nanotherapeutics for enhancing targeting
Mucus barrier in GIT	Mucus layer consisting of mucin	Mucus layer thickening in Mucinous cancer, Crohn’s disease Mucus layer thinning in ulcerative colitis	Fabrication of functionalized nanotherapeutics for increasing mucus adhesion or penetration
Immune environment in GIT	Epithelial barrier Immune response	Disrupted intestinal epithelial barrier in IBD, autoimmune disease, and infection Cell surface specific receptor expression and ROS overproduction in inflammation	Preparation of ligand-modified and microenvironment-responsive nanotherapeutics for enhancing targeting and controlled drug release
Gut microbiota	Diversity of gut microbes	Dysbiosis in gastrointestinal and liver diseases, metabolic diseases, immune-related diseases, and tumors	Preparation biomolecule (e.g. enzyme)-responsive nanotherapeutics for controlled drug release
Blood	Phagocytosis of MPS and RES Interference of PC	/	Fabrication of PEG-functionalized nanotherapeutics of cell membrane coated nanotherapeutics for prolonging circulation time in body

MPS: mononuclear phagocyte system; PC: protein corona; RES: reticuloendothelial system.

### 2.1. Biochemical-luminal barriers

The biochemical-luminal barrier, which includes variant pH values, sophisticated enzyme expression, peristalsis, and luminal fluid is a complex physiological environment in the GIT. For example, a large range of pHs is found in the GIT. The stomach’s parietal cells secrete hydrochloric acid, which keeps the pH low in the stomach (pH 1 to 2.5). In addition, from the proximal to the distal small intestine, the pH increases from 6.15 to 7.5. The pH decreases to 5.5 in the cecum; however, the pH increases from 6.87 in the proximal colon to 7.2 in the distal colon. External factors such as diet, drugs, microbial metabolism, and various diseases can also change the pH of the GIT (Ibekwe et al., 2008). Hypergastrinemia can decrease the pH in the stomach (Rehfeld, 2021); however, some drugs, such as H_2_ receptor antagonists and proton-pump inhibitors, can increases the pH in the stomach (Miller, 2018). In addition, microbial fermentation, bile acid metabolism of fatty acids, bicarbonate and lactate secretions, and intestinal volume and transit time can change the pH in the colon, which may result in low pHs (2.3 to 5.5) at sites of IBD (McConnell et al., 2008; Broesder et al., 2020). In addition to the variable pH, many enzymes, such as salivary amylase in the mouth, pepsin in the stomach, and lipase and amylase in the intestine, that are expressed throughout the GIT to aid in the digestion of food may lead to drug degradation (Zhang & Merlin, 2018). In addition, the transport time of drugs in the GIT is variable and influenced by many factors, such as fasting/food handling, sex, posture, age, diarrhea, and pathological status (e.g. irritable bowel syndrome or active ulcerative colitis) (Hebden et al., 2000; Arévalo-Pérez et al., 2020). Under normal conditions, the average transit times of drugs in the stomach, small intestine and colon are 1 ∼ 4 hours, 1 ∼ 6 hours, and 1 ∼ 3 days, respectively (Chu & Traverso, 2022).

The abnormal pHs in the GIT provide an opportunity for smart responsive drug delivery in diseased GITs. A variety of pH-responsive nanotherapeutics have been developed for the treatment of GIT diseases. Methacrylic acid copolymer (Eudragit^®^) is a synthetic cationic and anionic copolymer that is widely used as a pH-responsive carrier. The solubility of Eudragit at various pH values can be adjusted by changing the side group of the copolymer. Eudragit S100 (methacrylic acid and methyl methacrylate (1:2) copolymer), which can be dissolved in a pH 7.0 solution, is the most commonly used carrier for colon administration (Asghar & Chandran, 2006). Eudragit L100 (methacrylic acid and methyl methacrylate (1:1) copolymer) can be dissolved in a pH 6.0 solution, while Eudragit L100-55 (methacrylic acid and ethyl acrylate (1:1) copolymer) can be dissolved at pH 5.5 (Gao et al., 2021). Thus, Eudragit S100 can be combined in varying proportions with Eudragit L100-55 or Eudragit L100 to manipulate drug release in the pH range of 5.5-7.0 for the treatment of colon diseases (Khan et al., 1999). Chitosan, a cationic polymer, was also used as a pH-responsive carrier. Many amino groups exist in the chain of chitosan, which causes it to dissolve in low-pH solutions (Manna et al., 2023). Consequently, chitosan has been widely used as a pH-responsive carrier to deliver therapeutics to the stomach or small intestine (Cui et al., 2009).

Due to the short transit time in the stomach and small intestine, drugs have difficulty staying at diseased sites. Consequently, gastroretentive dosage forms (GRDFs) have been developed to extend the retention time of drugs at diseased sites through mucoadhesion, floating, magnetic, and sinking (high density) mechanisms (De Souza et al., 2021). Magnetic GRDFs use an external magnetic field to guide nanotherapeutics (with magnetism) to accumulate in the stomach or small intestine to prolong drug retention time (Bardonnet et al., 2006). Floating GRDFs usually use hydrophilic low-density materials as carriers and are administered by suspended nanotherapeutics in gastric juice. These GRDFs have been used for the treatment of *Helicobacter pylori* (*H. pylori*) infections (Yang et al., 2020a, Lopes-de-Campos et al., 2022). The results indicated that addition of a mucosal adhesion strategy can prolong the duration of nanotherapeutics exposures in the stomach to allow prolonged contact with bacteria.

### 2.2. Mucus barrier

The entire epithelial surface is covered with mucus, which is secreted by specialized cells called goblet cells. Mucus is the first line of defense against mechanical stress, pathogens, toxins, endogenous digestive systems, and microorganisms (Johansson, 2014). The main components of mucus are mucin (1-5%) and water (90-95%), as well as proteins, enzymes, lipids, and immune factors (Birchenough et al., 2015). Each part of the GIT has unique mucin, for example, mucin MUC5B exists in salivary glands and the esophagus, mucin MUC5AC and MUC6 are found in the stomach, and mucin MUC2 can be found in the small intestine and colon (Subramanian et al., 2022). In addition, the mucus layer in the small intestine is single, thin and loose (Dutton et al., 2019). In contrast, the stomach and colon have two layers of thick mucus, including the outer loose adhesive layer and the inner firm layer (Johansson et al., 2011). Mucins are negatively charged due to their extensive glycosylation domains (Cone, 2009). In addition, mucins contain regularly spaced hydrophobic domains, which increase the affinity for hydrophobic particles (Yang et al., 2014).

Diseases may change the composition, thickness, physical properties and function of mucus in the GIT. In some diseases, excessive and insufficient production of mucins causes a thicker and thinner mucus layer to form, which affects the function of the mucus. For example, the mucin MUC2 is overproduced in some mucinous cancers (Gundamaraju & Chong, 2021). In Crohn’s disease (CD), goblet cell hypertrophy and increased mucus production thicken the mucus layer, whereas in ulcerative colitis (UC), goblet cell depletion causes a marked reduction in mucus production and thinning of the mucus layer (Fernández-Tomé et al., 2021). Chronic inflammation or infection may change glycan profiles, which may alter the mucus charge and intestinal flora (Larsson et al., 2011).

The interactions between nanotherapeutics and mucus include mucus adhesion and active and passive mucus penetration (Naeem et al., 2015). Adhesion strategies between nanotherapeutics and mucus include electrostatic adsorption, hydrogen bonding, covalent interaction, hydrophobic interactions, van der Waals forces and polymer chain insertion (Maisel et al., 2015). Mucus has a negative charge; therefore, it electrostatically adsorbs with the positively charged nanotherapeutics. Chitosan is a cationic polymer, and the N-acetylglucosamine units of chitosan present a positive charge, which facilitates the electrostatic interaction of chitosan with mucus (TM et al., 2018). In addition, hydrogen bonding and hydrophobic effects also play key roles in influencing the ability of nanotherapeutics to adhere to mucus (Sogias et al., 2008). However, the adhesion of chitosan only occurred under acidic conditions (pH < 6). Chitosan is rich in hydroxyl and amino groups and can be chemically modified to form chitosan derivatives to enhance adhesion within a large pH range. For example, half-acetylated chitosan can be dissolved in neutral pH solution, and glycol chitosan is hydrophilic and can be dissolved in any pH solution (Sogias et al., 2008). In addition, pectin, alginate and its derivatives, cellulose derivatives, and polyacrylic acid are also used as adhesion materials in the mucus (Netsomboon & Bernkop-Schnurch 2016). Importantly, lectins can serve as specific adhesion materials for mucins (Villringer et al., 2018).

The outer layer of mucus regenerates quickly, and nanotherapeutics are easily removed from mucus only through adhesion. Consequently, mucus penetration is the key step in enabling nanotherapeutics to reach the epithelium. Poly (ethylene) glycol (PEG) is a hydrophilic and noncharged polymer that is widely used to modify nanotherapeutics to enhance mucus penetration. The diffusion rate of PEG-modified nanotherapeutics is 3 ∼ 10 times greater than that of unmodified nanotherapeutics (Cu & Saltzman, 2009). The molecular weight of PEG significantly influences the penetration of nanotherapeutics into the mucus. Nanotherapeutics coated with 2 ∼ 5 kDa PEG can diffuse rapidly in mucus; however, 10 kDa PEG-modified nanotherapeutics exhibit slow transport in mucus (Lai et al., 2009). Nanotherapeutics modified by PEG constitute a muco-insert permeation strategy that is realized by reducing the interaction between the surface and mucilage layer (Subramanian et al., 2022). The active mucus-penetrating system, also known as the mucolytic system, comprises mucus-lytic particles that can cut certain substructures within a three-dimensional mucus network. For example, disulfide breaking agents (such as N-acetylcysteine) and mucolytic enzymes (such as bromelain and papain) are used for fabricating mucolytic systems (Netsomboon & Bernkop-Schnurch, 2016). These systems do not destroy the entire mucus layer to maintain barrier function.

### 2.3. Gastrointestinal immune microenvironment (epithelium and lamina propria)

Gastric epithelial cells consist of surface foveolar mucus cells. In different parts of the stomach, specialized epithelial cells differentiate into parietal cells (which secrete hydrochloric acid and internal factors), chief cells (which secrete pepsin), and endocrine cells (Sáenz & Mills, 2018). The intestinal epithelium consists of a single layer of columnar intestinal epithelial cells (IECs), which include intestinal epithelial cells, goblet cells, Paneth cells, M cells, intestinal epithelial stem cells, and intestinal endocrine cells (Pelaseyed et al., 2014). Intestinal epithelial cells are the most abundant cells in both the small intestine and colon and form a physical barrier between the lumen and the host immune system. In the small intestine, they are responsible for the absorption of nutrients. The surface of the small intestine is covered with villi and several microvilli, which are used to increase the absorptive surface area (Andretto et al., 2021). In contrast, the colonic epithelium has no villi or microvilli but rather invaginates the intestinal glands. Goblet cells are secondary and promote the secretion of mucins to form mucus. Although goblet cells are present in both the small and large intestines, they are more abundant in the large intestine, where the mucus layer is thicker (Birchenough et al., 2015). Paneth cells are located at the base of crypts and are essential for intestinal immune homeostasis. They secrete antimicrobial granules and antimicrobial peptides (e.g. lysozyme, alpha-defensins, etc.) into the intestinal lumen. In addition, Paneth cells are important for stem cell maintenance (Huang et al., 2023). M cells are a special type of intestinal epithelial cells that are found mainly in the cellular layer of the small intestine and cover Peyer’s patches. The surface of M cells lacks microvilli and a mucus layer (Kobayashi et al., 2019). The delivery of luminal antigens to antigen-presenting cells (APCs) located in the subepithelial region is important in the intestinal immune system (Vitulo et al., 2022). The tight junctions between epithelial cells play an important role in maintaining structural integrity and regulating the permeability of the epithelial barrier. The junction molecules include junction adhesion molecules, occludin, claudin, and tricelluin (Zihni et al., 2016).

The intestinal immune system is large and complex and consists of GALT (gut-associated lymphoid tissue, including Peyer’s patches and isolated lymphoid follicles), the lamina propria, and the intestinal epithelium (Lee & Chang, 2021). Among them, Peyer’s patches, lymphoid follicles, and scattered APCs are the inductive sites of the immune response, while the lamina propria and intestinal epithelium are the effector sites (Marasini et al., 2014). IECs express pattern recognition receptors, which include Toll-like receptors, nucleotide-binding domain leucine-rich repeat containing receptors, RIG-I like receptors, and the C-type lectin family (Takeuchi & Akira, 2010). These receptors help IECs recognize and respond to microbes involved in the innate immune response. IECs also express major histocompatibility antigens, which act as APCs [54]. In addition, IECs also participate in adaptive immunity through T cells located in the basement membrane between epithelial cells (Ferguson, 1977). The lamina propria, another effector site of the immune response, contains a variety of immune cells, including B cells, T cells and innate immune cells, such as macrophages, dendritic cells (DCs), and eosinophils (Mowat & Agace, 2014). In addition, the extracellular matrix (ECM) is thought to be important for the regulation of immune responses. Biomolecules play important roles in controlling the adhesion, migration, proliferation, differentiation, and survival of immune cells (Daley et al., 2008).

In some pathological states, such as IBD (Qin, 2013), autoimmune diseases (Ilchmann-Diounou & Menard, 2020), and infection (Tong & Tang, 2017), the intestinal epithelial barrier is destroyed, leading to impaired epithelial integrity and intercellular junctions. This eEPR effect contributes to the passive targeting of small size nanotherapeutics to lesion sites (Hadji & Bouchemal, 2022). Moreover, epithelial damage leads to the accumulation of proteins with a positive charge, such as transferrin and eosinophil cationic proteins, which increase the adhesion of nanotherapeutics with negative surface charges to these proteins (Antoni et al., 2014). In this case, negatively charged coatings such as heparin (HEP) and hyaluronic acid (HA) were used to increase the accumulation of nanotherapeutics on the mucosa (Lee & Kamada, 2021). Luminal antigens activate immune cells; release cytokines and chemokines (such as IFN-γ, TNF-α, IL6, IL22, IL17, and CCL2) via epithelial PRRs (Neurath, 2014); and recruit additional immune cells into the intestinal mucosa, thus leading to adaptive immune system activation (Luissint et al., 2016). Similarly, the inflamed microenvironment alters the expression of specific receptors and cell adhesion molecules on the surface of colonic epithelial cells and immune cells, such as mannose receptors, transferrin receptors and folate receptors. Nanotherapeutics modified with corresponding ligands can achieve active targeting to deliver therapeutics to inflammatory cells (Liu et al., 2021). Moreover, inflammation is accompanied by the production of large amounts of ROS. Various gastrointestinal diseases, including gastrointestinal peptic ulcers, gastrointestinal malignancies, and IBD, are associated with ROS (Bertoni et al., 2020). High levels of ROS may cause cell damage and aggravate the inflammatory response (Bhattacharyya et al., 2014). Consequently, ROS-responsive nanotherapeutics have been developed to treat GIT diseases. The carrier can undergo a ROS-induced nonbreaking hydrophobic-hydrophilic transition (e.g. sulfur-containing compounds, selenium-containing compounds) or a ROS-induced structural dissociation (e.g. thioketal, phenylboronic acid/ester) to achieve ROS-responsiveness (Liu et al., 2020).

### 2.4. The gut microbiota

The gut microbiota plays an important role in the maintenance of health. The microbial density clearly increased from the proximal to the distal gut. In the stomach, although an extremely low-pH microenvironment inhibits the growth of bacteria, many microbes are found (Ruan et al., 2020). The majority of microbes in the stomach include *Proteobacteria*, *Firmicutes*, *Actinobacteria*, *Bacteroidetes*, and *Fusobacteria* (Bik et al., 2006). The amount and diversity of bacteria in the small intestine increases gradually from the duodenum to the jejunum to the ileum, and the bacterial densities in these regions are 10^3-4^ CFU/mL,10^3-7^ CFU/mL, and 10^9^ CFU/mL, respectively (Adak & Khan, 2019). Most of the gut microbiome is found in the colon, where the bacterial density is 10^12^ CFU/mL. The main bacterial phyla in the colon are *Firmicutes*, *Bacteroidetes*, *Proteobacteria*, and *Actinobacteria. Firmicutes* and *Bacteroidetes* account for approximately 90% of the total microbial composition (Santana et al., 2022). Gut microbes play important roles in metabolism, protective structure, and the nervous system (Adak & Khan, 2019). Gut microbes produce a series of enzymes that ferment dietary fiber and release short-chain fatty acids (SCFAs), such as acetate, propionate, and butyrate. These fatty acids are absorbed by the colon and provide approximately 10% of the body’s caloric needs (Duncan et al., 2007). Moreover, gut microbes and their metabolites also play an important role in regulating intestinal immunity. They can promote the differentiation of Treg cells, induce B cell production of IgA, and induce goblet cells secretion of mucins (Wu et al., 2017).

Dysbiosis (an abnormal microbiota composition) is defined as a change in the composition and diversity of the gut microbiome. Dysbiosis leads to changes in gut microbiota-related functions, such as changes in fermentation products; dysbiosis affects functions such as the maintenance of gut barrier integrity, and gut homeostasis (Khan et al., 2019). Dysbiosis may be associated with the development of various diseases, such as gastrointestinal diseases (e.g. IBD), liver diseases (e.g. fatty liver disease), metabolic diseases (obesity, diabetes), immune-related diseases, and tumors (Gomaa, 2020). In IBD, an increase in *Bacteroidetes* and *Proteobacteria* abundance and a decrease in *Firmicutes* abundance have been observed (Miquel et al., 2013), accompanied by a decrease in microbiome diversity (mainly a decrease in the relative abundance of *Firmicutes*) (Manichanh et al., 2006). In obese individuals, the proportion of *Bacteroidetes* decreases, and the level of *Firmicutes* increases (Liu et al., 2022).

An imbalance in the relative levels of pathogens and probiotics may contribute to the persistence of colitis (Kan et al., 2024), and probiotics as adjuvants are helpful for colitis treatment (Jakubczyk et al., 2020). However, the therapeutic effect of probiotics is limited because of the insufficient stability of probiotics in stomach and small intestine (Lopes et al., 2023). Nano-drug delivery system (NDDS) can avoid degradation of probiotics in stomach, and enhance targeting and prolong retention time in intestine (Zhang & Merlin, 2018). Natural polysaccharide have some biological activities such as anti-oxidation and anti-inflammation activities (Song et al., 2021). These natural polysaccharides are hardly digested in the mouth, stomach and small intestine due to lack of active carbohydrate enzymes (CAZymes) in these organs (Cui et al., 2021). However, polysaccharides can be transformed into multiple beneficial metabolites or fermentation products in colon since gut microbiota can encode multiple CAZymes in colon tissues (Cui et al., 2021). Based on this property, colon-targeting nanotherapeutics were prepared by using chitosan, alginate, HA and their derivatives as carriers (Li et al., 2022). These probiotics loaded nanotherapeutics were used to improve the intestinal microenvironment.

### 2.5. Intravenously injected nanotherapeutics

The intravenous administration of nanotherapeutics plays an important role in the delivery of therapeutics and imaging agents for the treatment of GIT diseases (Skotland et al., 2022). However, blood is a complex liquid with physiological barriers that may affect the stability and physical and chemical properties of nanotherapeutics. Proteins in the blood interact with charged nanotherapeutics and are adsorbed on the surface of nanotherapeutics to promote opsonization. These nanotherapeutics are easily taken up by the mononuclear phagocyte system (MPS) and reticuloendothelial system (RES), leading to rapid clearance from the blood (Owens & Peppas, 2006). MPS or RES phagocytosis not only limits the targeting efficiency of nanotherapeutics but also may enhance the accumulation and toxicological effects of nanotherapeutics in organs, such as the spleen, kidney, and liver (Gustafson et al., 2015). In addition, when proteins bind to nanotherapeutics, the surface of the nanotherapeutics will form a protein corona, which may change the size, dispersion, and stability of the nanotherapeutics, and further change the nano-biological interface, thus alerting the targeting effectiveness, immunogenicity, and intracellular toxicity (Moraru et al., 2020).

Based on the eEPR effect, nanotherapeutics may pass through the inflammatory vascular endothelium to accumulate at the lesion sites (Watanabe et al., 2016). To reduce the formation of protein coronas and prevent the rapid clearance of nanotherapeutics from the body, hydrophilic materials such as PEG are widely used as coatings to reduce the interaction between proteins and nanotherapeutics (Pozzi et al., 2014). PEG-functionalized nanotherapeutics are also called ‘stealth’ nanotherapeutics because they can create a barrier layer to block the adhesion of opsonins to nanotherapeutics, thereby allowing nanotherapeutics to escape from the RES and prolong the duration of systemic circulation (Owens & Peppas, 2006). Cell membrane camouflaged nanotherapeutics are a new type of biomimetic nanotherapeutics that can combine nanotherapeutics with the unique function of cell membranes. Red blood cells act as transport vehicles for blood circulation, and coating red blood cell membranes confers immune escape properties, thereby allowing for long circulation periods, which are essential for therapeutics delivery (Luk & Zhang, 2015). In addition, nanotherapeutics modified by specific proteins can also reduce clearance by RES to achieve active tissue targeting (Grafe et al., 2016).

## 3. Application of nanotherapeutics for the treatment of gastrointestinal diseases

### 3.1. H. pylori infection

*H. pylori* is a slightly aerobic, spiral, flagellate gram-negative bacterium. *H. pylori* mainly colonizes in the gastric epithelium below the mucus layer, which can produce a variety of virulence factors to cause gastric lesions. Gastric colonization by *H. pylori* is associated with urease, flagella, adhesion molecules and proteins on the surface of bacteria (De Falco et al., 2015), in which urease and flagella are considered as important factors (Perrais et al., 2014). *H. pylori* has been identified as the pathogen in human gastric mucosal infections and affects at least half of the world’s population. *H. pylori* infection may cause acute and chronic gastritis, gastric ulcers, peptic ulcers, and other gastrointestinal diseases (Yang et al., 2020b). Gastric cancer is the fifth most common malignant tumor and the third most fatal cancer in the world. Approximately 80% of gastric cancer cases are associated with *H. pylori* infections (Song & Zhou, 2015). Consequently, *H. pylori* was classified as a group I carcinogen by the International Agency for Research on Cancer (World Health Organization). *H. pylori* infection is also associated with neurological, dermatological, hematological, and other extra gastric diseases (Gravina et al., 2018).

Treatment of *H. pylori* infection mainly relies on a combination of antimicrobial and antisecretory drugs. The first-line treatment for *H. pylori* infection is standard triple therapy, which includes proton-pump inhibitors (PPIs) combined with two antibiotics (e.g. clarithromycin, amoxicillin, and metronidazole). However, antibiotic eradication rates are declining globally and are as low as 60% in some countries (Thung et al., 2016). The main reasons for eradication failure is increased resistance to antibiotics, particularly clarithromycin, metronidazole, and levofloxacin (Hu et al., 2017). The mechanisms of antibiotic resistance include modifications of targeting molecules, the enzymatic degradation of antibiotics, multidrug efflux pump systems, and biofilm formation (Ansari & Yamaoka, 2022). In addition, the short residence time of antibiotics in the stomach, the insufficient concentration of antibiotics, and the presence of a gastric mucosal barrier are also responsible for treatment failure (Lopes et al., 2014).

Nanotherapeutics have obvious advantages in the treatment of *H. pylori* infections. A NDDS can increase the stability of drugs by preventing acid or enzyme degradation in the stomach. In addition, NDDS can help therapeutics overcome gastric emptying, thereby increasing gastric retention time (Spósito et al., 2021) and penetration into the mucus layer (Lai et al., 2009). Furthermore, targeting the aggregation of nanotherapeutics at disease sites and the sustained release of therapeutics can increase drug concentration at target sites (Karthik et al., 2021). Due to the targeting ability of nanotherapeutics, the administered dose of therapeutics is reduced, and the side effects of drugs are decreased (Shimanovich & Gedanken, 2016). Nanotherapeutics may overcome the drug resistance of bacteria by destroying biofilms, oxidizing lipid or DNA, disrupting enzyme activity, causing plasmid damage, and other means (Zaidi et al., 2017). Lipid NPs, polymer NPs, protein NPs, inorganic metallic NPs, silicon dioxide NPs and biomimetic NPs have been explored for their ability to treat *H. pylori* infections. Among them, metallic NPs and polymer NPs have attacted an increasing amount of interest because of their special properties. In addition, emerging biomimetic NPs offer new possibilities for the treatment of *H. pylori* infections.

#### 3.1.1. Metallic NPs

Metallic NPs can be used as carriers to encapsulate antibacterial drugs or exhibit intrinsic antibacterial properties to treat *H. pylori* infections (Zhi et al., 2019). The antibacterial mechanisms of metallic NPs have been reviewed in several studies (Chen et al., 2014).

Silver NPs (Ag NPs) display excellent antibacterial activity by destroying bacterial cell membranes, inactivating urease or promoting ROS production (Franci et al., 2015). Both Ag NPs (Amin et al., 2012) and Ag NPs embedded in polymeric NPs (Almeida Furquim de Camargo et al., 2020) have been used for *H. pylori* infection treatment. For example, Kuo *et al.* established an *H. pylori* infection model based on the Mongolian gerbil model and prepared Ag NPs/clay complexes using silver ions, which significantly reduced *H. pylori* density *in vivo*, in a dose-dependent manner (Kuo et al., 2014). Amin *et al* designed a green synthetic pathway for the preparation of Ag NPs using the berry extract of *S. xanthocarpum*. The prepared Ag NPs had good stability and strong anti-*H. pylori* activity, even against multidrug resistant strains. In addition, Ag NPs also inhibited urease activity linearly (Amin et al., 2012). Experiments in *H. pylori*-infected rats verified that 16 mg/kg Ag NPs completely removed *H. pylori* from the stomach after seven days of treatment (Amin et al., 2014). All the evidence indicated that Ag NPs can effectively treat *H. pylori* infection in animal models.

**Figure 2.**
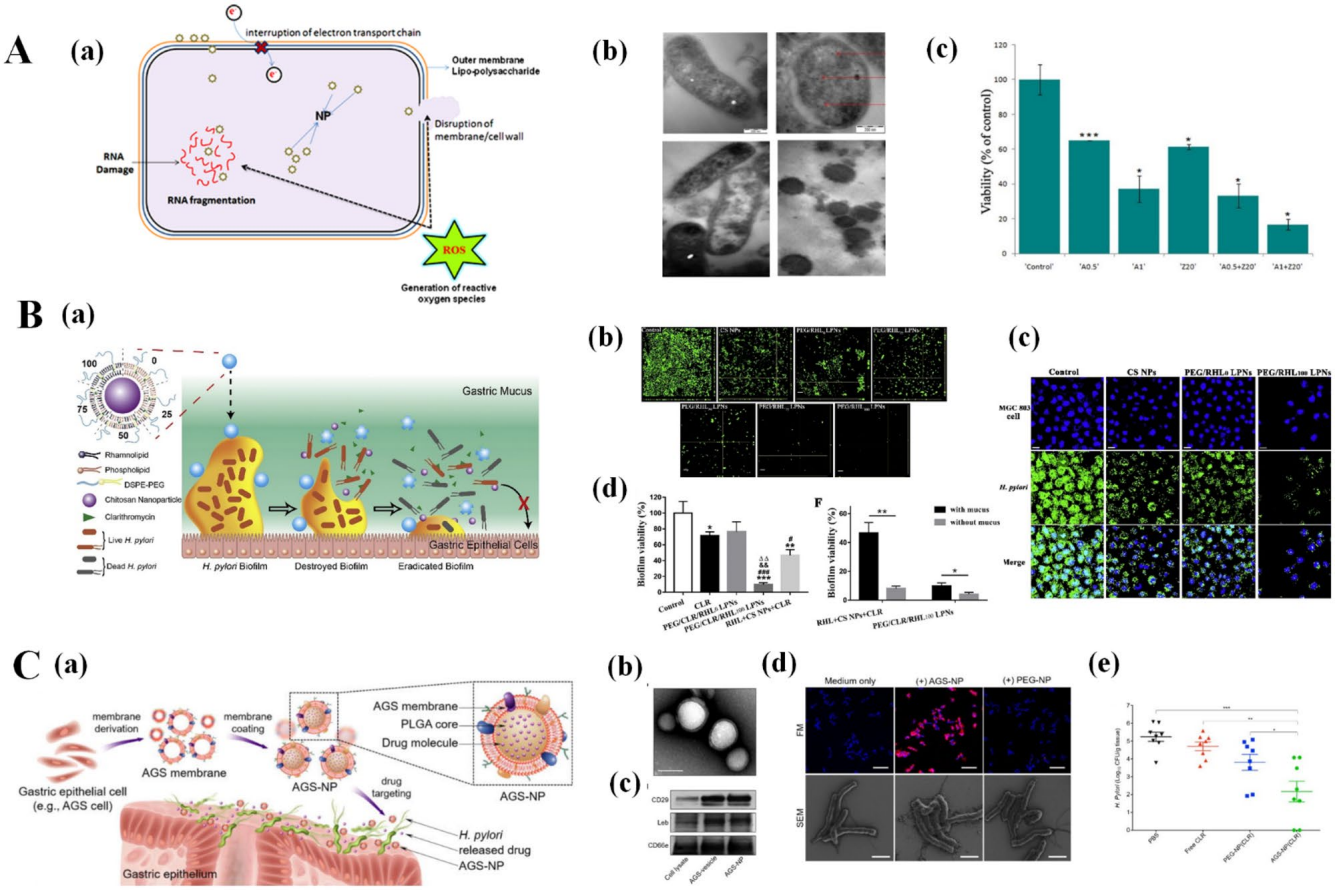
Nanotherapeutics used for *H. pylori* infection. (A) Effects of ZnO-PEI NPs on *H. pylori* infection. (a) Antimicrobial mechanism of ZnO-PEI NPs; (b) TEM images showing the internalization of the ZnO-PEI NPs into *H. pylori* and the morphological transition of *H. pylori*; (c) synergistic effect of the ZnO-PEI NPs and ampicillin. Adapted from Chakraborti et al. (2013) under the terms and conditions of creative commons attribution 4.0 international license license (https://creativecommons.org/licenses/by/4.0/). (B) Effects of lipid polymer NPs (LPNs) on *H. pylori* infection. (a) Schematic illustration of the structure of LPNs and the process of eradicating bacterial biofilm by LPNs; (b) inhibition of biofilms formation by LPNs; (c) inhibition of *H. pylori* adhesion to MGC 803 cells by LPNs. Blue fluorescence indicates MGC-803 cells. Green fluorescence indicates *H. pylori*; (d) The effect of LPNs on biofilm viability in the mucus barrier biofilm model; (e) comparison of the biofilm viability in PEG/CLR/RHL100 LPNs and the mixture groups in the model with or without mucus. Reprinted from Li et al. (2019) with permission. Copyright (2019), Elsevier. (C) Effects of membrane-coated NPs (AGS-NPs) on *H. pylori* infection. (a) Schematic illustrations of the preparation of AGS-NPs and their use for targeted antibiotic delivery to treat *H. pylori* infection; (b) TEM image of AGS-NPs stained with uranyl acetate; (c) Western blotting analysis of AGS membrane-specific protein markers; (d) FM and SEM images of AGS-NPs targeting *H. pylori* bacteria. Reprinted from Angsantikul et al. (2018) with permission. Copyright (2018), John Wiley & Sons, Inc.

ZnO NPs also displayed antibacterial activity against a variety of gram-positive and gram-negative bacteria with acceptable biosafety (Jones et al., 2008). ZnO NPs are highly toxic to bacteria but have poor toxicity toward normal cells (Reddy et al., 2007). Chakraborti *et al.* prepared polyethyleneimine (PEI)-functionalized ZnO NPs (ZnO-PEI NPs) for the treatment of *H. pylori* infection (Figure 2A). ZnO-PEI NPs were significantly internalized by bacterial cells, resulting in oxidative stress, increased ROS content, morphological changes, and rRNA degradation in *H. pylori* (Figure 2A,a). ZnO-PEI NPs exhibited moderate antibacterial activity (40% inhibition) at a safe concentration (20 μg/mL), but when the prepared NPs were combined with 1 μg/mL ampicillin, more than 80% inhibition of *H. pylori* was observed (Figure 2A,b–c) (Chakraborti et al., 2013). Attia *et al.* demonstrated that, compared with amoxicillin, ZnO NPs combined with amoxicillin significantly reduced *H. pylori* activity (Attia et al., 2022). In addition, plant extract-modified metallic NPs were also employed for the treatment of *H. pylori* infections to decrease the toxicity of metallic NPs (Amin et al., 2012).

#### 3.1.2. Polymer NPs

Polymer NPs have been widely used for disease treatment in recent years due to their good biocompatibility, biodegradability, and easy modification (El-Say & El-Sawy, 2017). Polymer NPs were also used for the treatment of *H. pylori* infection and exhibited enhanced targeting of the gastric mucosa (Graham & Dore, 2016). The strategies for targeting the gastric mucosa by polymer NPs include mucosal adhesion, mucosal penetration, floating rafts, and others (Shu et al., 2023). For example, Arif *et al.* prepared pH-responsive thiolated chitosan/poly (malic acid) NPs loaded with amoxicillin for the treatment of *H. pylori* infection. The biocompatibility, pH sensitivity, and adhesion properties of the NPs were confirmed *in vitro*. Importantly, amoxicillin-loaded NPs exhibited inhibition rate (77.1 ± 2.9%) of the growth of *H. pylori*, indicating that the antibiotic-loaded NPs had good antimicrobial activity (Arif et al., 2018). Biofilms are surface-attached communities of bacteria that are embedded in a self-produced hydrated matrix of extracellular polymeric substances. Biofilm formation is closely related to antibiotic resistance in bacteria (Hathroubi et al., 2018). Li *et al.* designed novel lipid polymer NPs that can inhibit *H. pylori* biofilm formation and overcome the mucus barrier in the stomach, thus providing a novel approach for the treatment of *H. pylori* infections (Figure 2B,a). The core-shell NPs were modified with DSPE-PEG_2000_ to increase the hydrophilicity of the NPs and aid in the penetration of the mucus layer of the stomach (Figure 2B,b–d). The prepared NPs inhibited *H. pylori* biofilm formation on biological surfaces (Li et al., 2019).

#### 3.1.3. Cell membrane-coated NPs

In recent years, cell membrane-coated NPs have been widely researched for antimicrobial therapy (Ma et al., 2022). Angsantikul *et al.* prepared gastric epithelial cells (AGS cells) coated with clarithromycin/PLGA NPs for *H. pylori* infection treatment (Figure 2C,a–c). Compared to PEG-coated clarithromycin/PLGA NPs, cell membrane-coated clarithromycin/PLGA NPs displayed enhanced targeting of *H. pylori* (Figure 2C,d). The reason may be that the key membrane proteins on AGS-NPs, including CD29 (integrin beta 1), blood group Lewis b (Leb), and CD66e (carcinoembryonic antigen-related cell adhesion molecule-5 or CEACAM5) can bind to *H. pylori*. Moreover, compared to those of PEG-coated clarithromycin/PLGA NPs, the cell membrane-coated clarithromycin/PLGA NPs signifcanltly decreased the bacterial content in the gastric tissues of mice, which indicated that cell membrane-coated clarithromycin/PLGA NPs can be effective against *H. pylori* infection (Angsantikul et al., 2018).

### 3.2. Inflammatory bowel disease

**Figure 3.**
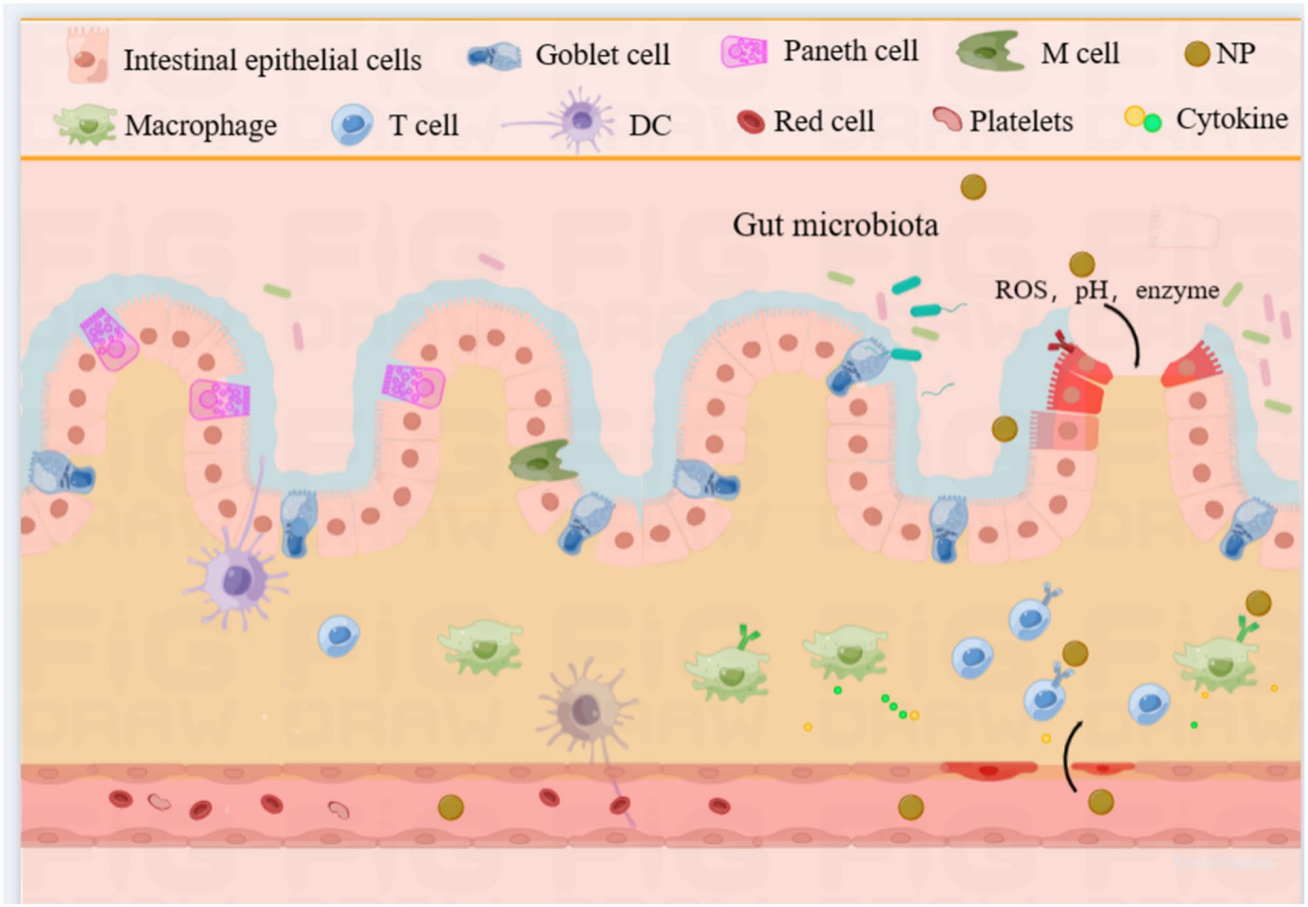
Structure of the Colon and the application of NPs in the Colon. Created by the author Shan Li.

IBD is a chronic and recurrent intestinal inflammatory disease that encompasses CD and UC (Lin et al., 2023). Both UC and CD cause chronic recurrent inflammation of the GIT, but they present with different pathological states. UC continuously and primarily affects the colon and rectum, however, CD usually discontinuously and predominantly affects the distal ileum and colon (Naeem et al., 2020). The pathogenesis of IBD is mainly related to the genetic predisposition of the individual and abnormalities in gut microbiota homeostasis (Colombel, 2014) (Figure 3). The early diagnosis and treatment of IBD are essential for preventing the development of severe disease and complications. The goal of treatment is to alleviate and control disease progression (Wright et al., 2018). Current medications for IBD include corticosteroids, 5-Aminosalicylic acid (5-ASA), immunomodulators, and biological agents.

#### 3.2.1. Passive targeting nanotherapeutics for IBD treatment

The passive targeting of nanotherapeutics to inflammatory sites is mainly determined by the physical and chemical properties of nanotherapeutic, such as their size, surface charge and morphology. According to the route of administration, nanotherapeutics can be delivered to inflamed sites in the colon through endothelial delivery via the blood and epithelial delivery via the luminal side of the GIT (Youshia & Lamprecht, 2016). Lamprecht *et al.* measured the adhesive ability of particles to the colon of trinitrobenzenesulfonic acid-induced ulcerative colitis rats with 0.1, 1, or 10 μm diameters. Among these particles, those 0.1 μm in diameter exhibited strong mucosal binding to the colon, and the number of particles adhering to the colon increased significantly with decreasing particle size (Lamprecht et al., 2001). GIT-associated nanotherapeutics are usually internalized by paracellular transport or ingested by gastrointestinal epithelial cells (Hua et al., 2015). Small nanoscale nanotherapeutics can easily adhere to mucus and diffuse in the mucus (Lautenschläger et al., 2014). Importantly, epEPR can allow small nanotherapeutics to pass through the damaged epithelium and more easily enter inflamed colonic tissue (Lamprecht, 2010). In addition, infiltrating inflammatory cells, such as macrophages and dendritic cells, can increase the uptake of nanotherapeutics.

The adhesion of nanotherapeutics with different charges to the mucosa seems to be related to colonic pathophysiology. Jubeh *et al.* analyzed the differences in adhesion of anionic, neutral and cationic liposomes in the colons of rats with dinitrobenzene sulfonic acid-induced colitis. They found that anionic liposomes adhered twice as much as neutral or cationic liposomes to inflamed colonic mucosa, whereas in healthy mucosa, cationic liposomes adhered 3 times more than neutral or anionic liposomes (Jubeh et al., 2004). The reason may be that the colonic mucosa is composed of negatively charged sulfate, sialic acid and mucin (Yasmin et al., 2022), which causes nanotherapeutics with a positive charge to easily adhere to the mucosal surface by electrostatic action. However, positively charged proteins such as transferrin and eosinophil granulocytes accumulate on the surface of the inflammatory mucosa of in IBD mice due to epithelial cell damage (Antoni et al., 2014), which is beneficial for the adhesion of negatively charged nanotherapeutics.

#### 3.2.2. Active targeting nanotherapeutics for IBD treatment

Although nanotherapeutics can passively target inflammatory sites, their efficacy should be improved. Interestingly, specific receptors and adhesion molecules are overexpressed on colonic epithelial and/or immune cells (Liu et al., 2021), which provides an opportunity to achieve active targeting to inflammatory tissues via the use of nanotherapeutics with corresponding ligand modifications (e.g. molecules, peptides, and antibodies). The nanotherapeutics available for the targeted treatment of IBD are listed in Table 2.

**Table 2.** Representative active targeting nanotherapeutics for the treatment of IBD.

Receptors	Expression Cells	Ligands	Carriers	Therapeutics	References
CD44	IEC and macrophages	HA	PLGA/Chitosan	siCD98/CUR	(Xiao et al., 2016)
			HA-DA	Budesonide	(Vafaei et al., 2016)
			PLGA/PVA- Chitosan	KPV	(Xiao et al., 2017)
			ES100/HA/Chitosan/HP-β-CD	FK506	(Cai et al., 2021)
			PLGA	CUR	(Hlaing et al., 2022)
			PLGA/DPPC/L-Arg-CO2	Pterostilbene	(Wei et al., 2022)
		CS	PLGA/DPPC/CG-CO2	siCD98/BBR	(Yang et al., 2022)
			PLGA/Chitosan	CUR	(Zhang et al., 2019)
			Zein	Magnolol	(Wang et al., 2021)
			PBAE-SA-PAPE	CUR	(Xu et al., 2022)
			N-2-HACC	CUR	(Xie et al., 2023)
Mannose receptor	Macrophages and DCs	Mannose	TPP/p (CBA-bPEI)-PEG	siTNF-α	(Xiao et al., 2013)
			trimethyl chitosan	siTNF-α	(Chu et al., 2015)
			NLCs	Budesonide	(Sinhmar et al., 2018)
			Trimethyl chitosan	miR146b	(Deng et al., 2019)
			CD/CMI-DE	Apremilast	(Sun et al., 2021)
			CDs-EP/Meth-Man- chitosan	Apremilast	(Shabana et al., 2022)
		KGM	AceKGM	CUR	(Wang et al., 2022)
MGL	Macrophages and DCs	Galactose	trimethyl chitosan–cysteine	siMap4k4	(Zhang et al., 2013)
			PLGA	siTNF-α	(Huang et al., 2018)
			PLGA	siTNF-α/IL-22	(Xiao et al., 2018)
Folate receptor	Macrophages	Folate	PLGA/PLA-PEG	6-shogaol	(Zhang & Xu, 2018)
			PLGA/PLA-PEG	17-AAG	(Yang et al., 2020a)
			Lecithin/DSPE-PEG	Cyclosporine A	(Li et al., 2023)
CD98	IEC and macrophages	CD98 Ab	PEI/UAC-PEG	siCD98	(Xiao et al., 2014)
		CD98 Fab’	PLA/bPEI-PVA/NHS-PEG-Mal	Quantum dots	(Xiao et al., 2014)
TfR	IEC and macrophages	7peptides	PEG-b-PCL	Coumarin 6	(Du et al., 2013)
LFR	IEC	Lactoferrin	CP/HA/LF	Rhein	(Luo et al., 2021)
F4/80	Macrophages	F4/80 Ab Fab’	PLA-PEG	siTNF-α	(Laroui et al., 2014)
PepT1	IEC and macrophages	KPV	PLGA-KPV/MMT/chitosan	Cyclosporine A	(Wu et al., 2019)
CCR5	Macrophages	CCL4	PLGA	Piceatannol	(Gong et al., 2021)
Dectin-1 receptor	Macrophages	β-glucan	Lentinan	Budesonide	(Lin et al., 2021)
MAdCAM-1	Vascular endothelial cell	MAdCAM-1 Ab	Manganese oxide	/	(Truffi et al., 2017)
			PLGA-PEG	Quantum dots	(Truffi et al., 2020)

HA: hyaluronic acid; CS: chondroitin sulfate; CUR: curcumin; DA: decylamine; PVA: poly(vinyl alcohol); KPV: lysine-proline-valine tripeptide; FK506: tacrolimus; BBR: berberine; DCs: dendritic cells; KGM: konjac glucomannan; MGL: macrophage galactose-type lectin; N-2-HACC: N-2-hydroxypropyl trimethyl ammonium chloride chitosan; TfR: transferrin receptor; LFR: lactoferrin receptor; PepT1: peptide transporter 1; CCR5: C–C motif chemokine receptor 5; CCL4: C–C motif ligand 4; MAdCAM-1: mucosal addressin cell adhesion molecule-1.

**Figure 4.**
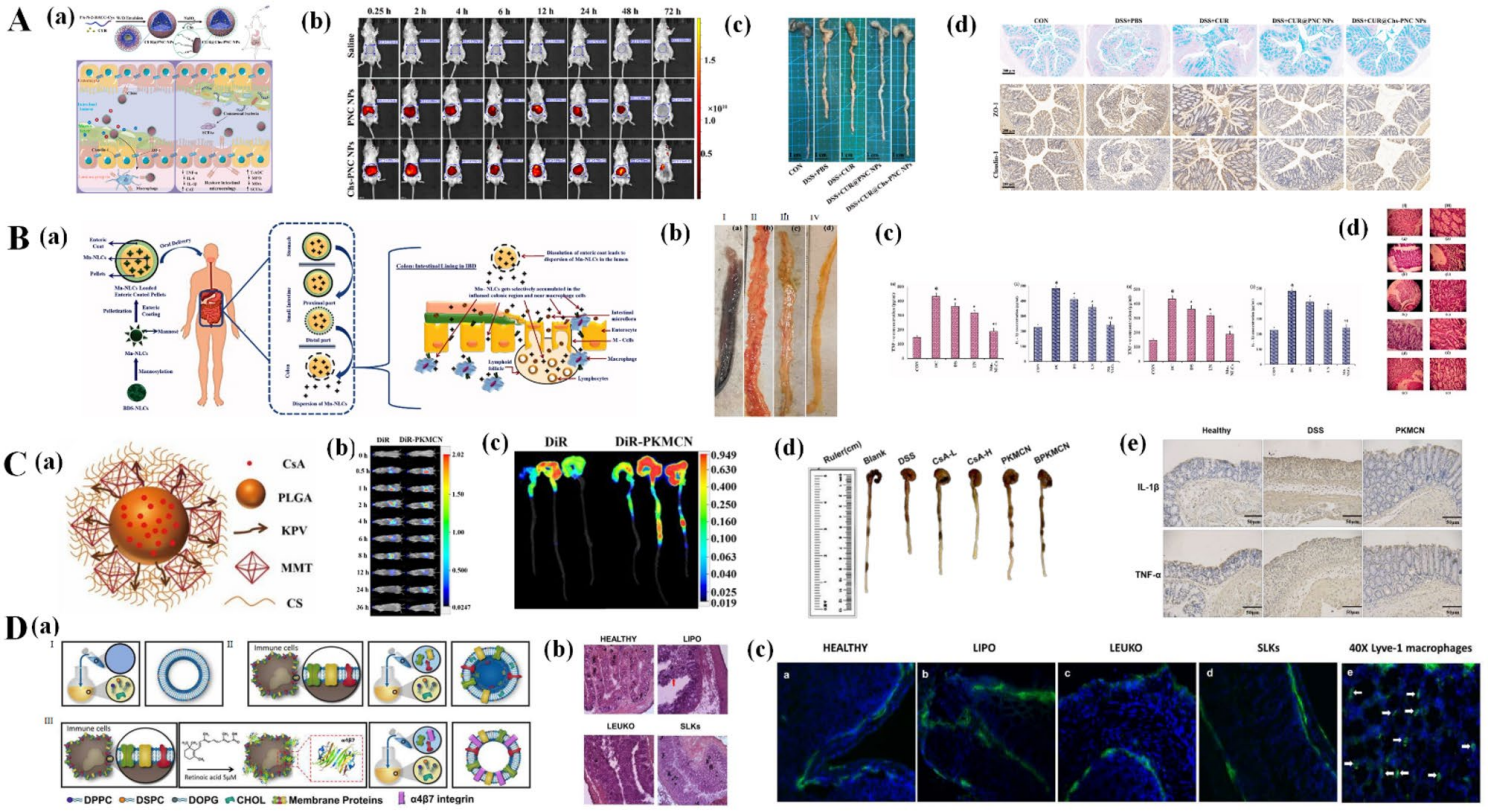
Targeting nanotherapeutics used for IBD treatment. (A) CD44 targeted NPs loading CUR for the targeting treatment of UC. (a) Schematic illustration of the CUR@Chs-PNC NPs for site-specific treatment of UC; (b) biodistribution of NPs in colitis mice after various administration time; (c) Representative images of the Colon; (d) HE staining results of different groups. Reprinted from Xie et al. (2023) with permission. Copyright (2023), Elsevier. (B) Mn-NLC for IBD treatment. (a) Schematic representation of the effect of budesonide-loaded Mn-NLC on IBD treatment; (b) gross morphology of the Colon at the end of the treatment period (*n* = 6); (c) tissue TNF-α and IL-1β levels, MPO activity and histopathological score. Reprinted from Sinhmar et al. (2018) with permission. Copyright (2018), informa UK limited. (C) Effects of KPV-modified nanoparticles (PKMCN) on IBD. (a) Schematic illustration of structure of PKMCN; (b) *in vivo* luminescence imaging of mice with DSS-induced colitis; (c) *ex vivo* luminescent images of colonic tissues after 24 hours of gavage; (d) Representative images of the Colon; (e) the immunohistochemical staining for IL-1β and TNF-α in Colon tissues. Reprinted from Wu et al. (2019) with permission. Copyright (2019), Royal Society of Chemistry. (D) Effects of specialized leucocyte bodies (SLKs) on IBD. (a) Nanoparticle assembly and characterization. Schematic representation of (I) liposomes, (II) leukosomes, and (III) specialized leukocyte assembly; (b) Histological analysis of colons; (c) immunofluorescence analysis of colons stained with CD45 (green) and DAPI (nuclei, blue). Reprinted from Corbo et al. (2017) with permission. Copyright (2017), Royal Society of Chemistry

CD44 is a transmembrane glycoprotein involved in the recruitment of leukocytes and the activation of immune cells during inflammation (Föger et al., 2000). CD44 is highly overexpressed on the surface of colonic epithelial cells and macrophages in colitis and is a major cell surface receptor of chondroitin sulfate (CS). In addition, HA, laminin, osteopontin, and serglycin can also serve as ligands of CD44 with high affinity (Li et al., 2021). Xei *et al*. designed CS-modified NPs (CUR@Chs-PNC NPs) loaded with CUR for the targeted treatment of UC (Xie et al., 2023). After oral administration, CUR@Chs-PNC NPs can be able to actively target intestinal epithelial cells via CD44-mediated targeting, and the accumulated NPs can modulate intestinal immune function (Figure 4A,a,b). In a colitis mice model, Cur@Chs-PNC NPs significantly reduced colonic shortening, decreased proinflammatory cytokine levels, and reduced oxidative stress with restoration of barrier function (Figure 4A,c–d).

The mannose receptor is overexpressed on the surface of macrophages and DCs at the IBD site and has a strong affinity for mannose (Van der Zande et al., 2021). Sinhmar *et al.* developed a mannosylation nanostructured lipid carrier system (Mn-NLCs) for the targeted delivery of budesonide (Figure 4B,a) (Sinhmar et al., 2018). In a colitis rat model, compared with nontargeting counterparts, Mn-NLCs significantly reduced the clinical activity score and improved colon shortening and morphology. In addition, Mn-NLCs reduced the levels of inflammatory cytokines (e.g. TNF-α and IL-1β) and myeloperoxidase activity in colon tissue. Histological results showed that targeting nanotherapeutics can promote the repair of colonic crypts and the healing of ulcers (Figure 4B,b–c). These results suggest that Mn-NLC can target inflammatory regions of the colon with good therapeutic efficacy.

The oligopeptide transporter Pept-1 is located on the brush border membrane of the intestine and is mainly responsible for the transport of protein digestion products (dipeptides and tripeptides) (Adibi, 1997). Pept-1 is not expressed in normal colonic epithelial cells, but is expressed on the membrane of chronically inflamed colonic epithelial cells and macrophages (Colas et al., 2017). The lysine-proline-valine (KPV) tripeptide has anti-inflammatory properties and high affinity for PepT1 (Dalmasso et al., 2008). Wu *et al*. developed a cyclosporine A (CyA)-loaded KPV-modified nanosystem (CyA-PLGA-kPV/MMT/Cs NPs, PKMCN) for colitis treatment (Figure 4C,a). Targeted nanotherapeutic accumulation in the inflamed region of the colon was 24-fold greater than that in healthy colon tissue (Figure 4C,b–c). Importantly, the targeting nanotherapeutics significantly improved the survival rate, colon length, weight loss and DAI score, and decreased the expression of inflammatory factors in mice with colitis (Figure 4C,d,e) (Wu et al., 2019).

Mucosal addressin cell adhesion molecule-1 (MAdCAM-1) is involved in the homing of α4β7 integrin-expressing T lymphocytes to inflammatory sites in the GIT (Lamb et al., 2018). MAdCAM-1 is highly expressed in the lamina propria and intestinal endothelial cells of patients with IBD and has recently been recognized as a marker of IBD (Souza et al., 1999). Corbo *et al*. engineered a bionic vesicle rich in integrin α4β7, called a specialized leucocyte body (SLK) (Figure 4D,a) (Corbo et al., 2017). These authors demonstrated that SLKs can target MAdCAM-1-overexpressing activated endothelial cells in inflamed colonic tissues and accumulate in the lumen of blood vessels. SLKs significantly relieved DSS-induced colon tissue and systemic inflammation in mice (Figure 4D,b–c). Importantly, in addition to exerting anti-inflammatory effects, bionic nanotherapeutics can improve vascular function and reduce colon tissue edema.

#### 3.2.3. Microenvironment responsive nanotherapeutics for IBD treatment

In addition to targeting strategies, the controlled release of therapeutics at inflammatory sites has also be studied. In IBD, the gut microenvironment changes greatly. These changes include the loss of epithelial barrier integrity, the production of numerous cytokines and ROS, and changes in the microbiota. Changes in the physiological characteristics of the GIT include a decrease in pH and transport time (Yasmin et al., 2022). A variety of microenvironment (e.g. pH, ROS, enzymes, and GSH) responsive nanotherapeutics have been developed for IBD treatment.

##### 3.2.3.1. pH-responsive nanotherapeutics

**Figure 5.**
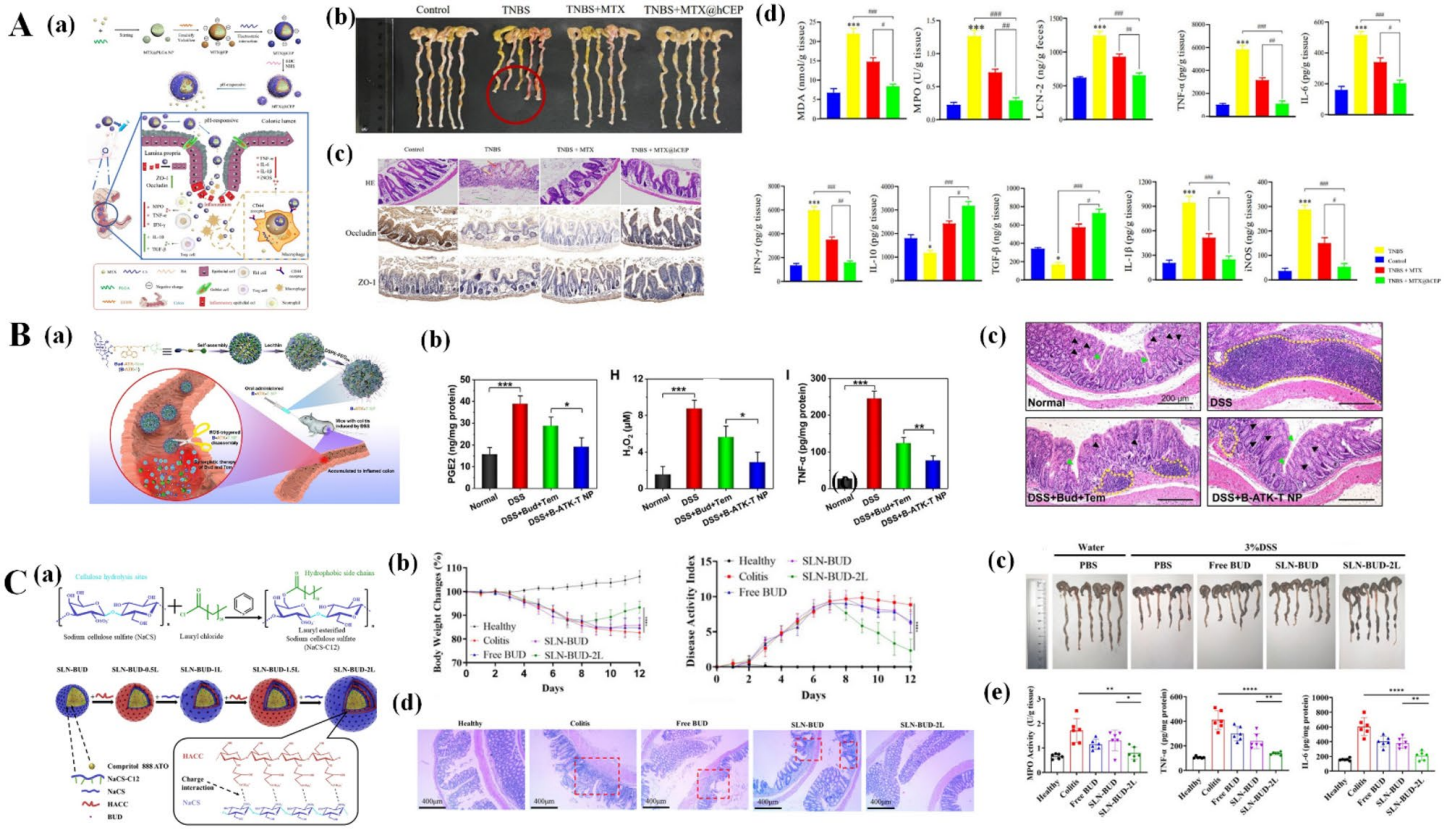
Microenvironment responsive NPs used for IBD treatment. (A) pH-responsive NPs used for IBD treatment. (a) Schematic illustration of MTX@hCEP taken orally for the treatment of TNBS-induced colitis in mice; (b) morphology of Colon length (*n* = 5); (c) H&E images of mouse Colon sections and representative immunohistochemical staining of occludin and ZO-1 in Colon sections; (d) levels of MDA, MPO, and LCN-2 activity and pro-inflammatory factors and anti-inflammatory factors. Reprinted from Lv et al. (2023) with permission. Copyright (2023), Elsevier. (B) ROS-responsive NPs (B-ATK-T NPs) for IBD treatment. (a) Schematic representation of the aromatized thioketal-bridged Janus prodrug nanoassemblies and ROS-responsive drug release at inflammatory sites; (b) fluorescence microscopy images showing the intracellular generation of ROS in cells; (c) the expression levels of representative factors of inflammation and oxidative stress in the Colon; (d) HE stained histological sections. Reprinted from Li et al. (2019) with permission. Copyright (2019), Elsevier. (C) Enzyme-responsive NPs for IBD treatment. (a) Synthesis of NPs; (b) mouse body weight changes and DAI score changes in each group after 12 days; (c) Colon photographs; (d) Histological evaluation of Colon tissue in each group; (e) average MPO activity and the expression of the proinflammatory cytokines TNF-α and IL-6 in each group. Reprinted from Zhang et al. (2023b) with permission. Copyright (2019), BioMed Central ltd.

Compared to that in the stomach and small intestine, the pH in the colon was greater even in patients with IBD. Eudragit and its derivatives can maintain certain stabilities in an acidic microenvironment (e.g. stomach) and can be degraded in a neutral or basic microenvironment. Consequently, these materials can serve as pH-responsive delivery syetems for IBD treatment by oral administration. Lv *et al*. described the use of Eudragit S100(ES100) material to encapsulate methotrexate (MTX) and fabricate pH-responsive nanotherapeutics (MTX@hCEP) (Figure 5A,a) (Lv et al., 2023). The *in vitro* release profile of MTX@hCEP in a simulated stomach, small intestine and colon media confirmed that these nanothrapeutics can maintain stability in an acid microenvironment and rapidly release their payload in normal or basic microenvronments. The authors also demonstrated that MTX@hCEP can accumulate in the colon in a TNBS-induced mouse model, which significantly relieves colitis symptoms, reduces the level of inflammation and maintains intestinal epithelial barrier function (Figure 5A,b–d). Trendafilova *et al*. compared the drug release properties of 5-ASA-loaded mesoporous silica NPs that were coated with Eudragit S100(anionic copolymer based on methacrylic acid and methyl methacrylate) or double coated with Eudragit S100 and Eudragir RL100 (a copolymer of ethyl acrylate, methyl methacrylate and a low content of methacrylic acid ester with quaternary ammonium groups). The authors found that tatol release of Eudragit S100 monolayer-coated NPs occurred within 1-2 hours in phosphate buffer (pH = 6.8). However, the combination of double-coated NPs with the Eudragir RL100 coating can be used to achieve total release in 4-5 hours, which can control the release profile and reduce burst release (Trendafilova et al., 2016).

##### 3.2.3.2. ROS-responsive nanotherapeutics

Activated immune cells (mainly macrophages and neutrophils) and IECs in the GIT produce large amounts of ROS, which are involved in the initiation and progression of IBD (Tian et al., 2017). ROS in the intestinal tissues of patients with IBD are significantly greater than those in normal tissues (Abed et al., 2022). Consequently, a variety of ROS-responsive materials, including boronic ester-containing, polyoxalate, and thioketal-containing materials, have been developed to fabricate nanoplatforms for IBD therapy (Dou et al., 2020). Li *et al*. synthesized a Janus prodrug (B-ATK-T) with ROS-responsive aromatized thioketal (ATK) in combination with budesonide (Bud) and the antioxidant tempol (B-ATK-T NPs) (Figure 5B,a). The authors demonstrated that B-ATK-T NPs can rapidly release Bud and tempol in H_2_O_2_-media. In colitis model mice, B-ATK-T NPs accumulated in colitis tissues, had reduced systemic exposure, and alleviated colitis symptoms and inflammation (Figure 5A,b–c) (Li et al., 2019). Based on the dual characteristics of increased ROS and decreased pH at colitis sites, pH/ROR dual-response nanotherapeutics are expected to increase therapeutic efficacy. Our team designed and synthesized a pH/ROS dual-responsive carrier (named CA-Oxi-αCD), which was formed by modifying cyclodextrin with cinnamaldehyde (which has anti-inflammatory activity) and the ROS scavenger 4-(hydroxymethyl) phenylboronic acid pinacol ester. The syn-sized materials were used as carriers to load cyclosporine A for the targeted treatment of colitis. The pH/ROS dual-responsive NPs could be internalized by activated macrophages and downregulate the expression of proinflammatory cytokines *in vitro. In vivo* experiments demonstrated that pH/ROS dual-responsive NPs accumulate in inflamed colon tissue, improve the symptoms of colitis, and reduce the levels of inflammatory factors and ROS (Li et al., 2023).

##### 3.2.3.3. Enzyme-responsive nanotherapeutics

Microbes in the gut secrete many enzymes that are responsible for the metabolism of carbohydrates and reducing compounds (Peng et al., 2021). Enzyme-responsive carriers can be degraded by specific enzymes in the colon, thereby allowing drugs to be released into the colon for therapeutic purposes. The mechanism of the enzymatic reaction involves mainly the reduction/oxidation of the substrate by the enzyme and the formation/cleavage of chemical bonds in the presence of the enzyme. Zhang *et al*. designed gut cellulase-responsive NPs with sodium cellulose sulfate as an enzyme substrate that were subsequently ntroduced to the surface of budesonide-loaded solid lipid nanoparticles (SLN-BUD-2Ls) (Figure 5C,a). The results showed that SLN-BUD-2L had an ideal bud release rate and cellulase responsiveness. This NP also had good therapeutic and anti-inflammatory effects in mice with DSS-induced colitis (Figure 5C,b–e) (Zhang et al., 2023b).

#### 3.2.4. Other emerging nanotherapeutics for IBD treatment

In recent years, nanotherapeutics with special properties have been used for the treatment of IBD, including biomimetic nanotherapeutics and gut microbiota-modulated nanothrapeutics.

##### 3.2.4.1. Biomimetic nanotherapeutics

In recent years, nanotherapeutics based on natural cell membrane (CM) has attracted more attention in the treatment of IBD. After coated with CM, nanotherapeutics have some biological characteristics similar to the source cells, such as low immunogenicity, long circulation time, tissue homing and biological barrier crossing properties (Han et al., 2024). For example, activated leukocytes are intrinsically capable of homing to the site of inflammation, consequently, leukocyte membrane-coated nanotherapeutics were employed for the targeting therapy of inflammatory diseases, such as IBD (Jin et al., 2018). In addition, macrophage membrane-coated NPs was also used to deliver rosiglitazone (RLZ) for the targeting treatment of DSS-induced colitis in mice. In vivo experiments demonstrated that macrophage membrane-coated nanotherapeutics could increase the targeting efficiencies and anti-inflammatory effects of nanotherapeutics. Furthermore, macrophage membrane-coated nanotherapeutics could protect mucosa by regulating the polarization of M1-type macrophages to M2-type (Sun et al., 2020). Membrane-coated nanozyme with superoxide dismutase (SOD) activities were also used for IBD treatment. For example, platelet membrane-coated nanozyme could hitchhike neutrophil to the site of inflammation and inhibit neutrophil aggregation, thus effectively reducing ROS levels and the expression of pro-inflammatory factors and restore the intestinal barrier (Yan et al., 2024). Platelet membrane-modification allows nanoenzymes easier to target inflammatory tissues than other uncoated nanoenzymes (Ma et al., 2022).

##### 3.2.4.2. Gut microbiota-modulated nanotherapeutics

The change of intestinal microflora is related to the development and progress of IBD (Lee & Chang, 2021). In IBD region, the composition and diversity of the gut microbiome is intricately associated with impaired intestinal epithelial barrier, immune activation, and various pathologies (Qiu et al., 2022). Consequently, gut microbiome-modulated nanotherapeutics were developed for IBD treatment, which can overcome biological and physical chemistry barriers of GIT, and act on commensal microbes in the intestinal lumen without intestinal epithelium penetration (Lee & Chang, 2021). For example, Lee et al. prepared a nanotherapeutics by conjugation of HA and bilirubin. The prepared nanotherapeutics can repair intestinal barrier function and significantly improve bacterial abundance and diversity in DSS-induced colitis mice, which provide a novel strategy for IBD treatment (Lee et al., 2020).

## 4. Conclusion and perspective

In this review, we emphasized the physiological and pathological microenvironment of the GIT. We also thoroughly reviewed the challenges and opportunities of nanotherapeutics for treating gastrointestinal diseases, including *H. pylori* infections and IBD but not gastrointestinal tumors due to the difference in microenvironments. Both passive and active targeted nanotherapeutics are widely used for treating gastrointestinal diseases. Alternatively, microenvironment-responsive nanotherapeutics have been employed for the treatment of gastrointestinal diseases Although nanotherapeutics are increasingly used to treat gastrointestinal diseases, including systemic or local drug delivery and oral vaccine delivery (Laroui et al., 2011), their clinical translation had been limited. Several factors, such as ong-term safety, complicated production, the storage of nanotherapeutics, and their limited efficacy compared to current therapeutics, need to be considered to achieve successful clinical translation. Importantly, the exploration of nanotherapeutics with efficacy and simple preparation methods is beneficial for achieving clinical translation.

## Data Availability

Data sharing not applicable.
